# Inflammatory Markers as Prognostic Factors of Survival in Patients Affected by Hepatocellular Carcinoma Undergoing Transarterial Chemoembolization

**DOI:** 10.1155/2017/4164130

**Published:** 2017-08-15

**Authors:** A. Rebonato, L. Graziosi, D. Maiettini, E. Marino, V. De Angelis, L. Brunese, S. Mosca, G. Metro, M. Rossi, G. Orgera, M. Scialpi, A. Donini

**Affiliations:** ^1^Department of Radiology, University of Perugia, Perugia, Italy; ^2^Department of General and Emergency Surgery, University of Perugia, Perugia, Italy; ^3^Department of Medical Oncology, University of Perugia, Perugia, Italy; ^4^Department of Medicine and Health Sciences, University of Molise, Campobasso, Italy; ^5^Department of Interventional Radiology, University La Sapienza, Rome, Italy

## Abstract

**Introduction:**

Transarterial chemoembolization (TACE) is a good choice for hepatocellular carcinoma (HCC) treatment when surgery and liver transplantation are not feasible. Few studies reported the value of prognostic factors influencing survival after chemoembolization. In this study, we evaluated whether preoperative inflammatory factors such as neutrophil to lymphocyte ratio and platelet to lymphocyte ratio affected our patient survival when affected by hepatocellular carcinoma.

**Methods:**

We retrospectively evaluated a total of 72 patients with hepatocellular carcinoma that underwent TACE. We enrolled patients with different etiopathogeneses of hepatitis and histologically proven HCC not suitable for surgery. The overall study population was dichotomized in two groups according to the median NLR value and was analyzed also according to other prognostic factors.

**Results:**

The global median overall survival (OS) was 28 months. The OS in patients with high NLR was statistically significantly shorter than that in patients with low NLR. The following pretreatment variables were significantly associated with the OS in univariate analyses: age, Child-Pugh score, BCLC stage, INR, and NLR. Pretreated high NLR was an independently unfavorable factor for OS.

**Conclusion:**

NLR could be considered a good prognostic factor of survival useful to stratify patients that could benefit from TACE treatment.

## 1. Background

Hepatocellular carcinoma, a highly insidious and prevalent tumor, is the sixth most common neoplasia and the third leading cause of cancer-related death worldwide [1]. Surgery is the treatment of choice for resectable HCC in an early stage of disease and in eligible patients with a good liver function.

Unfortunately, the intrahepatic recurrence rate is about 70% within 5 years after surgical resection. Most patients with HCC are diagnosed in an advanced stage when surgery and liver transplantation are not feasible; in this scenario, TACE is considered a good choice of treatment.

As a matter of fact, TACE, as demonstrated in the review written by Llonej and Bruix [2], showed a survival improvement compared to supportive care in patients affected by unresectable tumors. TACE can be offered to well-compensated patients with cirrhosis as a method to reduce their disease burden and potentially extend their life, as it is a reasonable and well-tolerated treatment with a minimal acceptable morbidity.

Few studies reported the value of prognostic factors influencing survival after chemoembolization. Recently, there are increasing evidences that systemic inflammation correlates with cancer patients' survival and prognosis. Studies have demonstrated that host inflammatory response to cancer cells is associated with tumor progression [3–9]. During the past decade, a variety of inflammatory factors have been identified as prognostic indicators of cancer -related survival.

Maltoni et al. [10] found that biological factors, such as leukocytosis, lymphocytopenia, and CRP, reached level B evidence-based recommendations of prognostic correlation in advanced cancer patients. In the last years, various markers of systemic inflammatory response including cytokines, neutrophil to lymphocyte ratio (NLR) and platelet to lymphocyte ratio (PLR) have been investigated for their prognostic roles in certain cancer patients [11]. In advanced gastrointestinal tumors, a high preoperative C-reactive protein level and high platelet count were frequently observed and were associated with poor patient prognosis [12]. The preoperative NLR also reflects patients' inflammation status, clinical stage, and patients' survival in colon cancer, lung cancer, and gastric cancer. Increased numbers of neutrophils and/or decreased numbers of lymphocytes may suppress lymphokine-activated killer cells, thereby increasing the propensity to metastasis.

Recently, the study of Jin et al. [13] demonstrated that baseline NLR and d-NLR may serve as convenient, easily measured prognostic indicators in advanced gastric cancer patients treated with preoperative chemotherapy and sequential R0 resection, especially to baseline NLR, which showed independent prognostic significance on RFS and OS. Also, the N/L ratio is considered an independent prognostic factor of survival in patients with gallbladder carcinoma as shown by Zhang et al. [14].

The NLR has been studied also in HCC patients who have undergone curative hepatic resection, radiofrequency ablation, and liver transplantation.

Based on the results of the recent study of Arai et al. [15], an elevated preoperative NLR is an independent predictive risk factor for patients undergoing a two-stage treatment with reductive surgery plus percutaneous isolated hepatic perfusion for multiple HCC with portal vein tumor thrombus.

In the study of Liao et al. [16], it was indicated that preoperative NLR, divided by X-tile for the cut point, is a simple prognostic marker for the patients with single-nodule HCC after curative surgical resection.

Only few studies have shown that an elevated pre-TACE NLR is associated with decreased survival.

A recent study done by Megan E. has demonstrated that periprocedural trends of serum NLR were associated with oncological outcomes in unresectable HCC undergoing TACE [17].

He assessed that serum NLR may be helpful to clinicians in providing prognostic information and monitoring response to therapy.

Xu et al. [18] demonstrated that preoperative NLR was an important and independent prognostic factor to predict the prognosis of 178 patients with intermediate HCC treated with TACE.

In this study, we evaluated whether preoperative inflammatory factors affected our patient survival outcomes who underwent TACE.

## 2. Methods

### 2.1. Patients

We retrospectively evaluated a total of 72 patients with hepatocellular carcinoma (HCC) who underwent TACE from January 2011 through December 2014 at the Radiology Department of our institution. Patients' records/information were anonymized and deidentified prior to analysis, so that an approval by the Ethic Committee was not necessary.

All the patients underwent either a CT scan or MRI in order to evaluate the extent of the disease. A multidisciplinary team decided the treatment to perform.

TACE was recommended for patients not eligible to undergo curative treatment including surgical resection, liver transplantation, and percutaneous ablation therapy. The eligibility criteria to select patients were as follows: absence of portal vein trunk involvement or extrahepatic metastasis; Child class A or B; platelet count > 60.000/mm^3^; and absence of ongoing spontaneous bacterial peritonitis.

**Figure 1:**
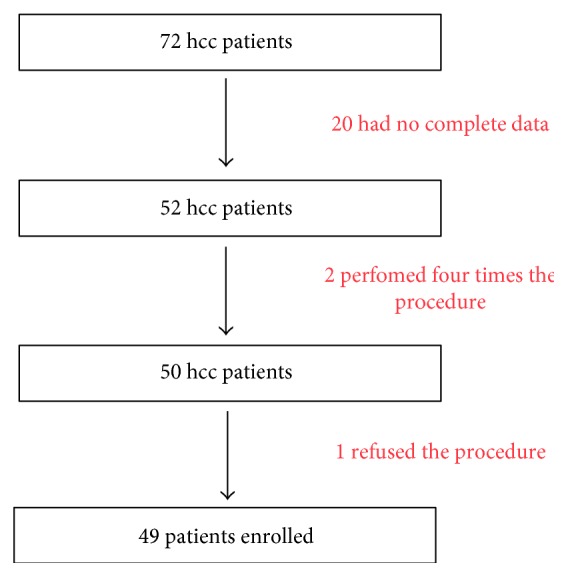
Flow chart of the patients enrolled.

We included, as shown in Figure 1, patients who underwent at least one TACE procedure and up to three procedures in three consecutive months. We excluded 20 patients because of incomplete data and medical history. The study population consisted of 49 patients, 39 men (80%) and 10 women (20%), with a median age of 75 years (range 49 to 88 years).

We enrolled patients with different etiopathogeneses of hepatitis: 18 hepatitis C virus, 19 criptogenetic, 10 alcohol related, and 2 hepatitis B virus. In 18 cases, HCC had a bilobar dissemination. Our patients had a different number of lesions: less than 3 lesions in 25 patients, between 3 and 5 lesions in 9 patients, and more than 5 lesions in 15 patients.

Thirty-eight patients had an A5 or A6 Child-Pugh class, and 11 had a B class. All patients had an intermediate BCLC stage and underwent TACE even if in Child A class, according to Milan criteria. Overall and NLR subgroup patients' characteristics are shown in Table 1.

### 2.2. TACE Technique

A 5-F introducer sheath was positioned in the common femoral artery. Selective catheterization of the hepatic artery was performed using 4- to 5-F catheters with different shapes and an 0.035^ hydrophilic guide wire (Glidewire, Terumo Europe, Leuven, Belgium); then a microcatheter (Progreate, Terumo Europe, Leuven, Belgium) was inserted coaxially. Superselective catheterization of the main feeders was performed whenever possible. The DEB-TACE protocol used DC beads (100–300 and 300–500 *μ*m; Biocompatibles, Surrey, UK) loaded with 50–75 mg of epirubicin hydrochloride. The loaded beads were mixed with iodinate contrast medium and saline to a ratio of 8 : 2 to optimize visualization during the infusion procedure. The embolization endpoint was determined by occlusion of HCC-feeding arteries; when not achieved, Spongostan pledgets were injected to obtain complete stasis.

### 2.3. Follow-Up

A CT or RMN was performed at 1 month from the procedure to assess radiographic response to TACE and 6 months thereafter.

The overall survival time (OS) was calculated from the date of the first TACE procedure to the date of death provided by the regional registry office, or at the conclusion of the study, end of June 2015. The progression free survival (PFS) was determined as the time lapse between the procedure and the progression of the disease defined according to mRECIST criteria [19]. Follow-up imaging was available at our institution only for 39 patients who were selected to evaluate the PFS; median PFS of all patients was 12 months.

### 2.4. Data Collection and Blood Samples

Demographic details, drug used during TACE, and survival data were prospectively collected using clinical records into a database. One venous blood sample was taken the day before the procedure and collected in an ethylenediaminetetraacetic acid-containing tube according to other studies present in the literature. The numbers of WBCs and platelets were determined with a hemocytometer. Absolute counts of particular cells were calculated by multiplying the percentage of particular cells by the number of WBCs. Patients were dichotomized on the basis of a median cutoff value, classifying high and low groups, and survival curves were analyzed for NLR and PLR. Preoperative NLR and PLR were calculated as the neutrophil/platelet count divided by the lymphocyte count. The patients were dichotomized at the median value of NLR, PLR, albumin, bilirubin, Child-Pugh class, and the presence or absence of ascites. Median value was recalculated when analyzing subgroups.

Data are collected on the approval of our Institutional Ethics Committee.

### 2.5. Statistical Analysis

Patients' descriptive analysis was generated, and their differences were investigated using Student *t*-test for quantitative data; for qualitative data, we used Fisher's exact test or chi-square test.

To compare OS and PFS between groups, the cumulative survival proportions were calculated using the product limit method of Kaplan-Meier, and differences were evaluated using the log-rank test. Only variables that achieved statistical significance in the univariate analysis were subsequently evaluated in the multivariate analysis using Cox's proportional hazard regression model. A *p* value of less than 0.05 was considered statistically significant. All statistical analyses were performed using the MedCalc Statistical Software version 14.8.1 (MedCalc Software bvba, Ostend, Belgium).

## 3. Results

The overall study population was dichotomized in two groups according to the median NLR value of 2.03; a high NLR group of 25 patients and a low NLR one of 24 patients.

Group demographic characteristics are summarized in Table 1.

Patients with high NLR had no significant difference in demographic characteristics according to the BCLC stage, age, Child-Pugh stage, etiology, number of lesions, and tumor size. Bilobar spread represented 75% of the high NLR group and 50% of the low NLR group. Lymphocyte count was significantly lower in the high NLR group while neutrophil count was higher than in the low NLR.

The PFS in patients with high NLR was not significantly worse than that in the low NLR group. Only creatine (*p* = 0.0350) and GFR (*p* = 0.0429) were associated with the PFS in univariate analyses. No pretreatment factor correlated with PFS in multivariate analyses. PFS analyses are resumed in Table 2.

**Figure 2:**
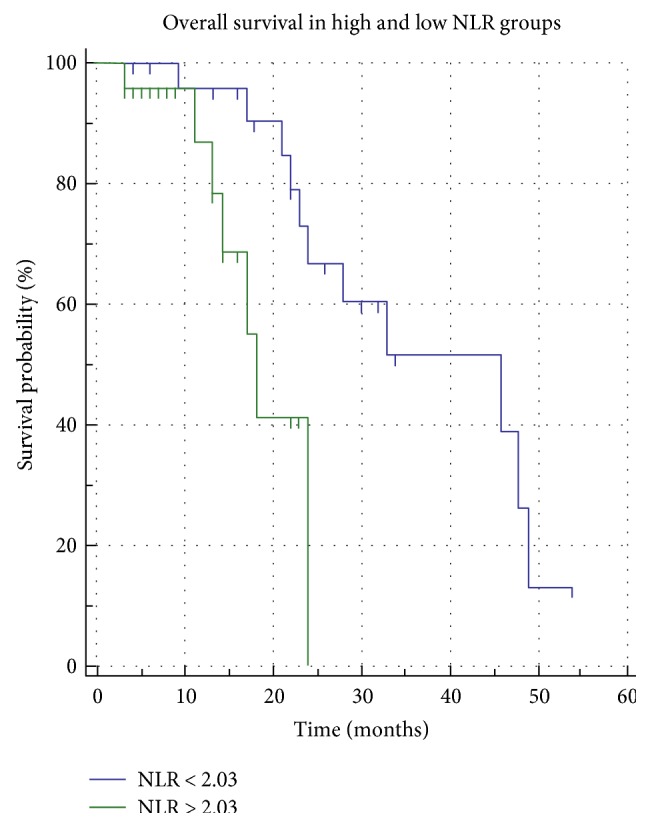
Kaplan-Meier curves showing a significant statistical difference in the OS between the high and low NLR groups (*p* = 0.0429).

**Figure 3:**
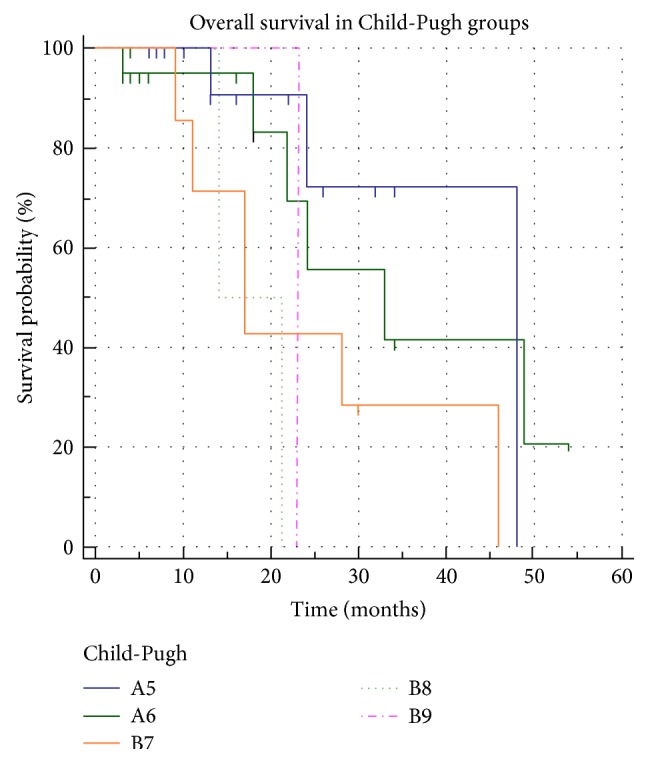
Kaplan-Meier curves showing a significant statistical difference in OS between Child-Pugh groups (*p* = 0.0365).

**Figure 4:**
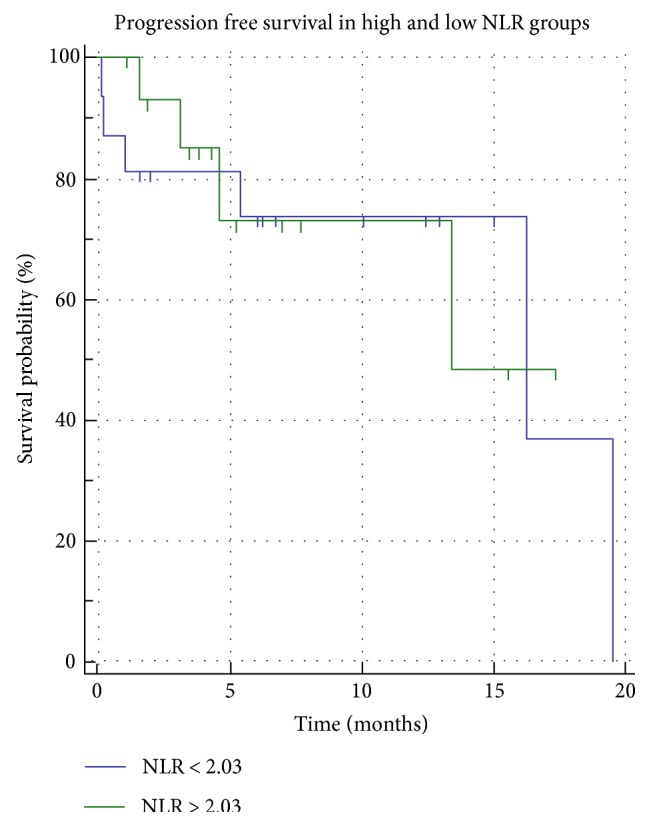
Kaplan-Meier curves showing a nonsignificant statistical difference in PFS between the high and low NLR groups (*p* > 0,05).

The median OS of all patients was 28 months. The OS in patients with high NLR was shorter than that in patients with low NLR (*p* = 0.0429) (Figure 2). The median OS in patients with high NLR was 18 months, and it was significantly worse than that in patients with low NLR (46 months). The following 5 of the 17 pretreatment variables were significantly associated with the OS in univariate analyses: age (*p* = 0.0325), Child-Pugh score (*p* = 0.0365) (Figure 3**)**, BCLC stage (*p* < 0.0199), INR (*p* < 0.0218), and NLR. Pretreatment of high NLR was an independent unfavorable factor for OS (HR = 11.5283; *p* = 0.0028) as well as INR (HR = 16.9893; *p* = 0.0193). PLR did not correlate with the PFR nor the OS (Figure 4). OS analyses are resumed in Table 3.

**Figure 5:**
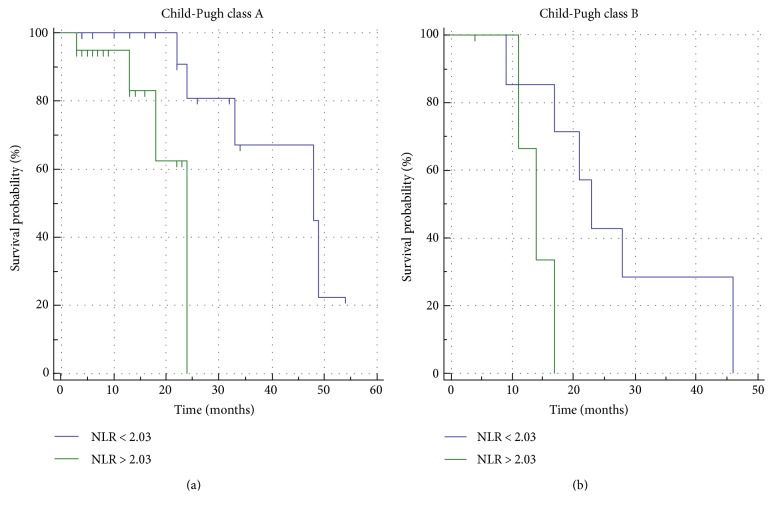
Kaplan-Meier curves showing a significant statistical difference between the high and low NLR patients in the Child-Pugh A (a) and B (b) class subgroups.

We dichotomized the two subgroups Child-Pugh class A and B using the NLR median value of 2.03; the high NLR group demonstrated to have a significant worse survival in both Child-Pugh class A (*p* = 0.0135) and B (*p* = 0.0437) (Figures 5(a) and 5(b)).

## 4. Discussion

The majority of patients affected by HCC have unresectable disease at presentation, and transarterial chemoembolization (TACE) has been widely used in these cases. In this study, we showed that the simple assessment of inflammatory markers before TACE is highly predictive of the outcome independently from the cirrhosis' cause. NLR and PLR are associated with overall survival in patients with unresectable HCC initially treated with TACE. Specifically, patients with unresectable HCC have worse outcomes when NLR or NPR is elevated before TACE. In fact, patients with NLR superior to 2.03 have a median survival of 14 months, and patients with PLR less than 2.03 have a median survival of 20 months.

Since Virchow's study [20], inflammation has been found to play an important role in the pathogenesis and progression of malignant tumors, including HCC [21–23]. Inflammation promotes tumor angiogenesis, invasion, and tumoral progression due to an altered regulation of T lymphocytes and chemokines. In addition, patients with a lymphocytopenia may have a higher risk of tumor recurrence and a worse prognosis [24]. In HCC patients, increase has been reported to be associated with a lower recurrence rate and better prognosis [25].

On the other hand, neutrophil stimulation caused an expression of various cytokines, such as interleukin 8 (IL-8), which is the core of the inflammatory and immune responses, and it results in tumor progression and metastasis [26].

Neutrophils promote tumor growth and metastasis by secreting vascular endothelial growth factor, angiopoietin-1, and matrix metalloproteinase-9. This angiogenic activity resulting from neutrophilia could increase the metastatic power of tumor.

A recent meta-analysis written by Xiao et al. [27] provided a strong evidence that the elevated NLR is prognostically significant in patients with HCC treated by either curative or palliative methods. To date, only few studies correlated inflammatory markers to prognosis of unresectable HCC patients addressed to TACE.

Various studies aimed at developing a simple validated prognostic score based on different predictive factors (scoring system, number and size of hepatic lesions, AFP value, and cirrhosis pathogenesis) of survival and TACE response in patients with HCC treated with TACE [1]. In his study, Wenzhe et al. demonstrated that high NLR and PLR were both associated with poor prognosis and metastasis in recurrent HCC patients treated with TACE, but high PLR was a better predictor of a 1-year OS. He showed other prognostic variables as independent unfavorable factors: vascular invasion and multiple tumors [28].

In our study, NLR demonstrated to correlate with survival even in patients with the risk already assessed according to Child-Pugh. We hypothesize that NLR and PLR may be useful in predicting survival-stratifying patients at high recurrence risk after TACE. In this way, it allows to make a stricter follow-up in these patients. In particular, for patients with abnormal elevation of NLR and/or PLR before TACE, it may be reasonable to add systemic or biologic therapies to TACE hoping to achieve better outcomes.

Compared to other prognostic markers, NLR and PLR seem to be a convenient predictor for HCC patients. It is low cost, reliable, easily obtainable, and repeatable. The limitations of the current study arise from factors that could affect the NLR and PLR of patients with unresectable HCC before TACE. Viral hepatitis could affect the hymmunestatus compared to those with HCC who are not infected with hepatitis virus. Level of blood neutrophils and platelets could be reduced by cirrhosis-associated hypersplenia.

Other limitations are related to the retrospective design of the study and the sample size of studied patients. However, the utility of these inflammatory parameter markers should be further validated and compared to treatment-naive patients.

Further prospective studies are necessary to confirm and expand our preoperative prognostic score model for patients with unresectable HCC. A definitive cutoff value of NLR, based on future large-sample study, is recommended.

## 5. Conclusion

In view of these findings, perhaps, HCC patients with high NLR may benefit from anti-inflammatory treatment. We hope that future research could test this hypothesis.

## Figures and Tables

**Table 1:** Overall, high and low NLR subgroups patients' characteristics and statistical differences between the two subgroups.

	Mean (min-max) or frequencies *n* = 49			
	Overall	Low NLR	High NLR	*p* value
Sex (male/female)	39/10	16/9	23/1	0.0106^∗^
Age (years)	75 (49–88)^┼^	75 (49–88)^┼^	74 (51–86)^┼^	0.477^∗∗^
Etiology (HCV/Cripto/Alcol/others)	18/19/10/2	11/9/3/2	7/10/7/0	0.2102^∗∗^
Child-Pugh class (5/6/7/8/9)	17/21/7/3/1	8/10/5/1/1	9/11/2/2/0	0.6081^∗∗^
Neutrophil (/mm^3^)	3485 (811–9130)^┼^	2389 (811–6019)^┼^	4533 (1920–9130)^┼^	0.002^∗∗∗^
Lymphocyte (/mm^3^)	1321 (360–2970)^┼^	1553 (759–2970)^┼^	1110 (360–1977)^┼^	0.0107^∗∗∗^
Neutrophil/lymphocyte	3.25 (0.61–19.02)^┼^ Median 2.03	1.5 (0.61–2.03)^┼^	5.06 (2.19–19.02)^┼^	0.0004^∗∗∗^
Platelet (/mm^3^)	115.857 (51.000–243.000)^┼^	111.041 (51.000–243.000)^┼^	117.000 (53.000–188.000)^┼^	0.6607^∗∗∗^
Platelet/lymphocyte	99 (33–391)^┼^	75.43 (33.69–142.83)^┼^	124.40 (52.50–391.78)^┼^	0.0048^∗∗∗^
BCLC stage (B/C)	34/15	17/8	17/7	0.9244^∗∗^
Tumor size (<5/>5 cm)	23/26	13/11	10/15	0.4796^∗∗^
Number of tumors (<3/3–5/>5)	25/9/15	15/4/6	10/5/9	0.4293^∗∗^
Extrahepatic spread (absent/present)	49/0	25/0	24/0	1.0^∗∗^
Bilobar spread (absent/present)	18/31	12/13	6/18	0.1697^∗∗^
Ascites (absent/present)	37/12	20/5	17/7	0.6791^∗∗^
Albumin (g/dL)	3.70 (2.51–4.80)^┼^	3.55 (2.51–4.46)^┼^	3.83 (2.77–4.80)^┼^	0.7460^∗∗∗^
Bilirubin (mg/dL)	1.04 (0.08–3.07)^┼^	0.96 (0.08–2.42)^┼^	1.11 (0.32–3.07)^┼^	0.6570^∗∗∗^
INR	1.18 (0.92–1.68)^┼^	1.20 (0.92–1.68)^┼^	1.16 (1.00–1.40)^┼^	0.1409^∗∗∗^
Creatinine (mg/dL)	0.90 (0.45–2.28)^┼^	0.84 (0.45–1.80)^┼^	0.95 (0.57–2.28)^┼^	0.2198^∗∗∗^
GFR (mL/min)	96.56 (29.00–197.00)^┼^	100.09 (29.00–197.00)^┼^	93.63 (30.00–146.00)^┼^	0.4283^∗∗∗^

^∗^Fisher's exact test; ^∗∗^*χ*^2^-test; ^∗∗∗^Student's *t*-test; ^┼^min-max; BCLC = Barcelona Clinic Liver Cancer; Cripto = criptogenetic; GFR = glomerular filtration rate; HCV = hepatitis C virus; INR = International Normalized Ratio; NLR = neutrophil to lymphocyte ratio; PLR = platelet to lymphocyte ratio.

**Table 2:** Pretreatment factors affecting progression free survival.

		*n*	mPFS	Univariate *p*^∗^	Hazard ratio (95% CI)	Multivariate *p*^∗∗^
Sex	Male	39	10	0.4581		
	Female	10	12			

Age (years)	>76	25	15	0.9328		
	<76	24	6			

Etiology	HCV	18	10	0.1911		
	Cripto	19	6			
	Alcol	10	17			
	Others	2	20			

Child-Pugh class	5	17	12	0.5016		
	6	21	6			
	7	7	15			
	8	3	—			
	9	1	—			

NLR	>2.03	25	8	0.4403		
	<2.03	24	12			

PLR	>87.99	25	7	0.3800		
	<87.99	24	14			

BCLC stage	B	34	10	0.1457		
	C	15	14			

Tumor size (cm)	>5	26	12	0.9600		
	<5	23	11			

Number of tumors	<3	25	12	0.7320		
	3–5	9	5			
	>5	15	10			

Extrahepatic spread	Absent	49	12	—		
	Present	0	—			

Bilobar spread	Absent	18	10	0.8673		
	Present	31	12			

Ascites	Absent	37	14	0.1823		
	Present	12	10			

Albumin	>3.7	25	8	0.2176		
	<3.7	24	15			

Bilirubin	>0.84	25	8	0.0841		
	<0.84	24	16			

INR	>1.18	25	15	0.5610		
	<1.18	24	10			

Creatinine	>0.78	25	5	0.0350	3.4639 (0.7593–15.8025)	0.1104
	<0.78	24	15			

GFR	>94.5	25	17	0.0429	0.6264 (0.1437–2.7311)	0.5356
	<94.5	24	5			

^∗^Log-rank test; ^∗∗^Cox's proportional hazards regression model.

BCLC = Barcelona Clinic Liver Cancer; Cripto = criptogenetic; GFR = glomerular filtration rate; HCV = hepatitis C virus; INR = International Normalized Ratio; mOS = median overall survival; mPFS = median progression free survival; NLR = neutrophil to lymphocyte ratio; PLR = platelet to lymphocyte ratio.

**Table 3:** Pretreatment factors affecting overall survival.

		*n*	mOS	Univariate *p*	Hazard ratio (95% CI)	Multivariate *p*
Sex	Male	39	46	0.1285		
	Female	10	23			

Age (years)	>76	25	49	0.0325	0.4838 (0.1267–1.8480)	0.2908
	<76	24	23			

Etiology	HCV	18	24	0.9782		
	Cripto	19	46			
	Alcol	10	—			
	Others	2	28			

Child-Pugh class	5	17	48	0.0365	0.9823 (0.1690–5.7078)	0.9842
	6	21	33			
	7	7	17			
	8	3	14			
	9	1	23			

NLR	>2.03	25	18	0.0429	11.5283 (2.3361–56.8918)	0.0028
	<2.03	24	33			

PLR	>87.99	25	24	0.9737		
	<87.99	24	33			

BCLC stage	B	34	48	0.0199	1.8542 (0.4128–8.3281)	0.4229
	C	15	23			

Tumor size (cm)	>5	26	24	0.6515		
	<5	23	33			

Number of tumors	<3	25	24	0.2565		
	3–5	9	17			
	>5	15	46			

Extrahepatic spread	Absent	49	28	—		
	Present	0	—			

Bilobar spread	Absent	18	46	0.6596		
	Present	31	24			

Ascites	Absent	37	48	0.0964		
	Present	12	23			

Albumin	>3.7	25	—	0.1210		
	<3.7	24	22			

Bilirubin	>0.84	25	23	0.2664		
	<0.84	24	33			

INR	>1.18	25	22	0.0218	16.9893 (1.6017–180.2098)	0.0193
	<1.18	24	—			

Creatinine	>0.78	25	24	0.7856		
	<0.78	24	24			

GFR	>94.5	25	21	0.7606		
	<94.5	24	24			

BCLC = Barcelona Clinic Liver Cancer; Cripto = criptogenetic; GFR = glomerular filtration rate; HCV = hepatitis C virus; INR = International Normalized Ratio; mOS = median overall survival; NLR = neutrophil to lymphocyte ratio; PLR = platelet to lymphocyte ratio.
